# Modulation of Locomotion and Reproduction by FLP Neuropeptides in the Nematode *Caenorhabditis elegans*

**DOI:** 10.1371/journal.pone.0135164

**Published:** 2015-09-25

**Authors:** Yan-Jung Chang, Tina Burton, Lawrence Ha, Zi Huang, Adewale Olajubelo, Chris Li

**Affiliations:** 1 Department of Biology, City College of New York, City University of New York, New York, New York 10031, United States of America; 2 The Graduate Center, City University of New York, New York, New York 10031, United States of America; University of Würzburg, GERMANY

## Abstract

Neuropeptides function in animals to modulate most, if not all, complex behaviors. In invertebrates, neuropeptides can function as the primary neurotransmitter of a neuron, but more generally they co-localize with a small molecule neurotransmitter, as is commonly seen in vertebrates. Because a single neuron can express multiple neuropeptides and because neuropeptides can bind to multiple G protein-coupled receptors, neuropeptide actions increase the complexity by which the neural connectome can be activated or inhibited. Humans are estimated to have 90 plus neuropeptide genes; by contrast, nematodes, a relatively simple organism, have a slightly larger complement of neuropeptide genes. For instance, the nematode *Caenorhabditis elegans* has over 100 neuropeptide-encoding genes, of which at least 31 genes encode peptides of the FMRFamide family. To understand the function of this large FMRFamide peptide family, we isolated knockouts of different FMRFamide-encoding genes and generated transgenic animals in which the peptides are overexpressed. We assayed these animals on two basic behaviors: locomotion and reproduction. Modulating levels of different neuropeptides have strong as well as subtle effects on these behaviors. These data suggest that neuropeptides play critical roles in *C*. *elegans* to fine tune neural circuits controlling locomotion and reproduction.

## Introduction

Neuropeptides are commonly used to modulate behaviors in both vertebrates and invertebrates. They can act synaptically within a neural circuit, as well as extra-synaptically to affect more distant neural circuits. These modes of action, in conjunction with the sheer number of neuropeptides, increase the diversity of behavioral outputs in an organism. Furthermore, a single neuropeptide gene may encode many peptides with similar or different amino acid sequences; these peptides may bind the same or multiple receptors with different affinities to exert different physiological activities. Hence, understanding the full range of neuropeptide activities has been challenging in any organism.

Data mining of the DNA sequence of the nematode *Caenorhabditis elegans* has revealed over 100 genes that encode neuropeptides [1,2,3,4,5,6,7,8,9,10]. The neuropeptides have been classified into three major groups: the insulin-related peptides (INS), the FMRFamide-related peptides (FLPs), and all other neuropeptides (NLPs). Signaling through these peptides affects numerous behaviors, including response to environmental conditions, metabolism, locomotion, and egg laying [3,4,5, 11,12,13,14,15,16,17,18,19,20,21]. We have been examining the role of FMRFamide-related peptides or FLPs in *C*. *elegans*. At least 31 *flp* genes encode FLPs and the expression patterns of many of these genes have been characterized [9,14,15,22]. Because the synaptic connectivity of all 302 neurons has been determined in *C*. *elegans* [23], the behavioral circuits underlying many behaviors, such as locomotion and reproduction, are well described.

Locomotion and reproduction represent two critical behaviors for *C*. *elegans* survival and propagation. *C*. *elegans* moves in a sinusoidal waveform when on a solid surface or in a liquid medium. This movement is mediated by sets of motor neurons residing in the ventral nerve cord and innervating dorsal and ventral body wall muscles [23]. Muscle contractions are mediated by the cholinergic A-, B-, and C-type neurons, including the VA, VB, VC, and AS neurons that innervate ventral muscles and the DA and DB neurons that innervate dorsal muscles [23,24,25,26]. Muscle relaxation is mediated by the GABAergic D-type neurons, DD and VD, which innervate dorsal and ventral muscles, respectively [23,27]. Both the DD and VD neurons express *flp-11*, while only the DD neurons express *flp-13* [22]. No *flp* expression has been identified in the A- and B-type neurons thus far. The locomotory motoneurons receive input from several command interneurons and together the neurons coordinate the ventral and dorsal muscles to generate the waveform for efficient forward and backward movement. These interneurons receive direct and indirect input from sensory neurons that transduce environmental stimuli. *flp-1* and *18* are expressed in a subset of the locomotory command interneurons [22].

The neural circuit for egg laying is also well characterized. The egg-laying muscles are innervated by the serotonergic and cholinergic HSN [26,28] and VC neurons [26], which receive input from several interneurons, including AVF and BDU, and the mechanosensory neuron PLM [23]. Similar to the locomotory circuit, the motor, sensory, and interneurons involved in egg laying express several *flp* genes, including *flp-19* in the HSN neurons, *flp-4* in the AVF interneurons, *flp-10* in the BDU interneurons, and *flp-20* in the PLM mechanosensory neuron [22,23]. Although the VC neurons are FMRFamide-like immunoreactive [29], the specific *flp* gene responsible for this immunoreactivity has not been identified. In addition, the neural circuit that regulates the number of eggs present in the uterus at any given time is unknown.

Because the locomotory and egg-laying circuits are well defined, we decided to explore how FLP neuropeptides modulate these two behaviors. We isolated deletion mutants in 11 *flp* genes, many of which are expressed in the locomotory or egg-laying circuits and some of which are not explicitly expressed in any neurons of the motor circuits, and examined how the locomotory and egg-laying behaviors were affected in these mutants. Most mutants displayed locomotory and/or egg-laying defects, suggesting that many FLP neuropeptides play a role in modulating these behaviors.

## Materials and Methods

### Maintenance of strains

Strains were maintained at 20°C as described [30]. The wild-type strain is N2 var. Bristol [30]. Strains used include: LGII: *flp-4(yn35)* [31]; LGIV: *flp-10(pk367)*, *flp-9(yn36)*; LGIV: *daf-10 flp-1(yn2)* [11]; LGV: *flp-21(pk1601*, *ok889)*, *flp-6(pk1593)*; LGX: *flp-18(tm2197)*, *flp-12(n4902)*, *flp-20(pk1596)*, *flp-3(pk361)*, *flp-19(pk1594)*, *flp-8(pk360)*. Transgenic lines include: *flp-3*: *ynEx179*, *ynEx180 [flp-3; myo-2*p::*GFP]; flp-4*: *ynEx178*, *ynEx231*, *ynIs99* [*flp-4; myo-2*p::*GFP]; flp-10*: *ynEx169*, *ynEx170*, *ynIs92*, *ynIs93*, *ynIs94*, *ynIs95*, *ynIs98 [flp-10; myo-2*p::*GFP; sur-5*::*GFP]; flp-19*: *ynEx171*, *ynEx232 [flp-19; myo-2*p::*GFP]; flp-20*: *ynEx167*, *ynEx168 [flp-20; myo-2*p::*GFP; sur-5*::*GFP]; flp-21*: *ynEx172*, *ynEx181*, *ynEx233 [flp-21; myo-2*p::*GFP]*. Integrated lines were not mapped and only *ynIs94*, *ynIs95*, *ynIs98 [flp-10; myo-2*p::*GFP]* were outcrossed before behavioral analysis.

### Isolation of deletion mutants

Libraries of mutagenized animals [32] were screened by polymerase chain reaction (PCR) with primers flanking the coding region of the different *flp* genes. Populations showing a deletion in the *flp* gene were sib selected until the *flp* deletion mutant was isolated. The *flp-18(tm2197)* mutant was generously donated by the Japanese National BioResource Project. Deletion mutants were backcrossed at least three times into a wild-type background to remove unlinked mutations.

### PCR and generation of transgenic animals

PCR was used to confirm the genotype of different strains. The program used was: 94°C for 1 minute, followed by 35 cycles of 94°C for 40 sec, 58°C for 40 sec, and 72°C for 40 sec to 1 min. To generate transgenic lines, the genomic region for representative *flp* genes was amplified, the product gel purified, and the purified product co-injected with the transgenic markers *sur-5*::GFP [33] and/or *myo-2*p::GFP [34]. The primer pairs used for amplification of genomic regions, sizes of promoter regions, co-injection markers, and concentrations of injected DNA are listed in Table 1. All of the 5’ primers used to amplify genomic regions were the same as the primers used in the promoter constructs to determine *flp* gene expression patterns [22], except for *flp-10*, where an additional 67 bp was used in the promoter region for the genomic fragment. Extrachromosomal arrays in transgenic lines were integrated into the genome as described [35] with slight modifications.

**Table 1:** Amplification of genomic fragments and co-injection markers for microinjection.

Gene	5’ primer sequence (5’ to 3’)	3’ primer sequence (5’ to 3’)	Length of region upstream of translational ATG (bp)	Length of region down-stream of stop (bp)	Concen-tration of micro-injected DNA (ng/μl)	Co-injection marker^	Concen-tration of co-injection marker (ng/μl)
*flp-3*	ACCCATTCGTTTTGGCAAACG	TGACTTGGTGTTGCACAGCTG	1995	1616	1	myo-2p::GFP	66
*flp-4*	TGTAGTACGTGACTGTAGCCC	CAAGGGTCATTCTCATTGTTAGCC	2943	1135	4.5	myo-2p::GFP	66
*flp-10*	CTAGTGTTGCTTCGCGATTC	CGCTTCGAGATCAAATCTTCG	1671	261	20	myo-2p::GFP & sur-5::GFP	80 & 500 ng/μl
*flp-19*	GACTCACCGTAGTAATCC	GTAGTTTGTCTTCCATCTACC	2920	509	35–70	myo-2p::GFP	88
*flp-20*	GGAAACATTGGTCGGGAGATG	CGCACATCGTTCGATTAG	3069	156	17	myo-2p::GFP & sur-5::GFP	80 & 500
*flp-21*	GCCCATCATGTACAGCCC	CCCGAATGCTGAATTGACCAAAC	2975	875	57	myo-2p::GFP	88

*myo-2*p::*GFP* [34] & *sur-5*::*GFP* [33]

### Behavioral assays

For all behavioral assays, healthy (i.e., non-starved, non-dauered) fourth larval stage animals were plated and used the following day as day 1 adults, except for egg retention assays, when animals were used two days later as day 2 adults. At least 40 animals of each mutant strain and at least 30 animals for each transgenic strain were assayed over multiple trials. All assays were performed at room temperature. Because of the inherent variability among animals and in scoring, multiple, independent researchers scored mutants and the composite averages were tallied.

#### Swimming assay

Animals were placed into 50 μl of M9 physiological buffer and the number of body bends made in 15 seconds was counted (as modified from [36]). Each individual animal was tested three times and the three values were averaged to give a single mean value for the animal.

#### Serotonin-induced inhibition

The presence of serotonin inhibits locomotion [37]. Animals were placed into 12.9 mM serotonin in M9 physiological buffer and the number of body bends made in 15 seconds was counted. Each individual animal was tested three times and the three values were averaged to give a mean value for the animal.

#### Egg-laying rate

Animals placed into physiological buffer are transiently inhibited from egg laying. This inhibition is overridden by the presence of serotonin. Animals were placed into 50 μl of 12.9 mM serotonin in M9 physiological buffer [28] and the number of eggs laid after 60 minutes was counted.

#### Egg retention

Animals were placed into 50 μl of a weak hypochlorite solution (1.2 ml of Chlorox bleach, 0.5 ml of KOH, water to 10 ml) to dissolve the adult mother. The number of eggs, which are resistant to the hypochlorite solution, was counted.

### Statistical analysis

All statistical analyses were performed using Prism software (GraphPad). Wild-type animals and mutants were compared using the Mann-Whitney test. Mutants and transgenic mutants containing a genomic transgene were compared as a group with a one-way ANOVA and Neuman Keuls posthoc test.

## Results

**Fig 1:**
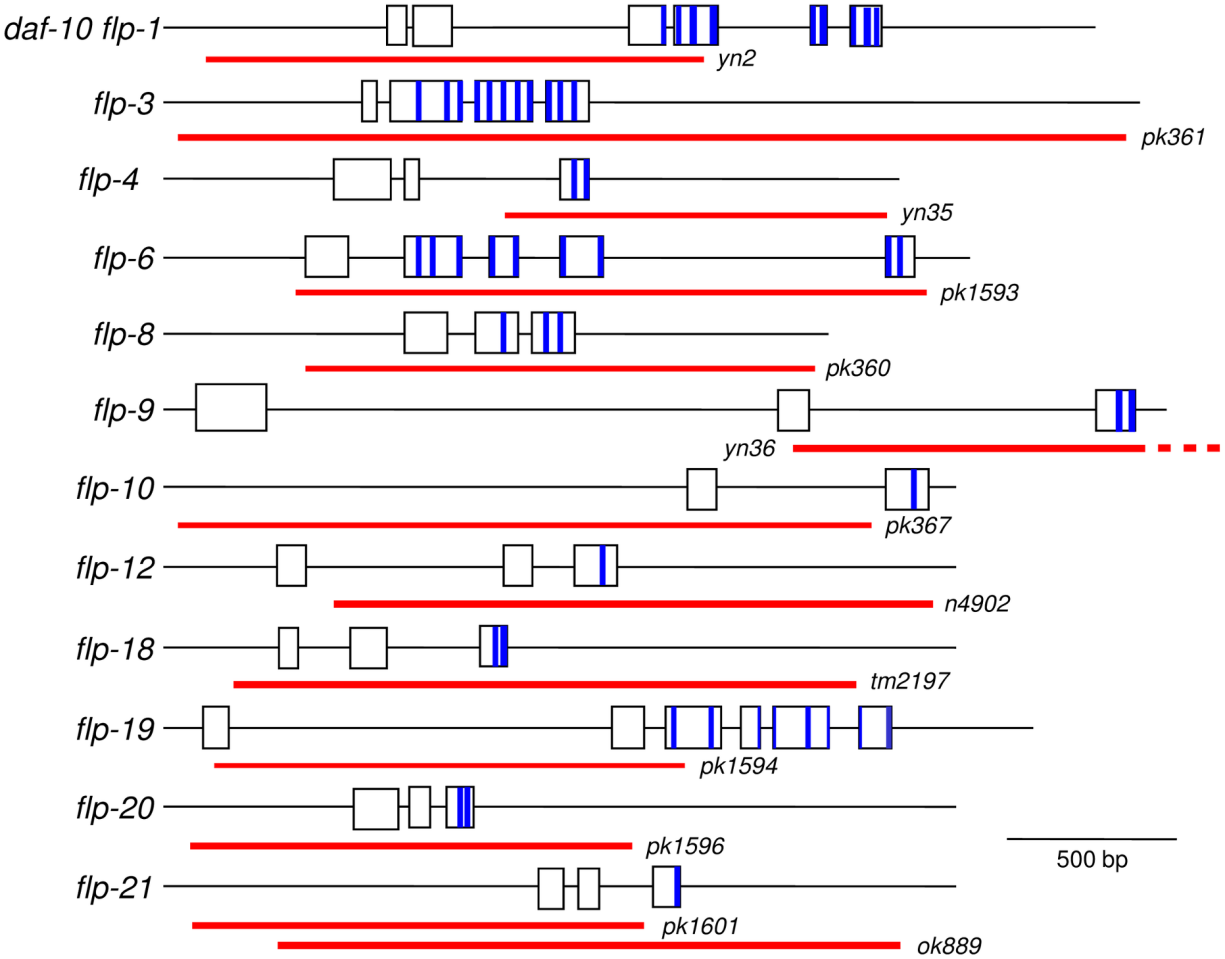
Genomic organization of the *flp* genes. The extent of the deletions are indicated below by the red bars. The peptide coding regions (not to scale) are indicated by blue bars; one peptide coding region may extend over two exons. Flanking sequences and sizes of the deletions are as follows: *daf-10 flp-1(yn2)* (CTAAATAATTTTAAAACGTA/CTTACCTTTCAAAGTTTGCA) [11], *flp-3* (GCACAGCTGGAGGGTGGAGG/CAAACGATTACTATTGTGTC; 2632 bp deletion), *flp-4* (TTCTGAAAAACTTTTAATAA/AGCTCGCCGAGCCGAGTCTTG; 928 bp deletion) [31], *flp-6* (CAAAAAAGCGAGTCGGGGGT/TCGTTTTTCACTTATTGAAC; 1662 bp deletion), *flp-8* (AGATAAATCTCAGAACAAGC/ATAATTTTGGCGTCATAGTT; 1414 bp deletion), *flp-9* (ATAATAATTATCTAGAAATA/GTACTTTCGATTCGATTCCT; 1881 bp deletion), *flp-10* (GCTTATTCTGCGTTCATCA/GTAAAAACTATCTTAATAAT; 1884 bp deletion), *flp-12* (CACTAGACATAGCTCTCCATG/TCAATTTGAATTTCTGAATA; 1732 bp deletion), *flp-18(tm2179)* (CGGAGCACTCCCGGCATTTC/GAGCGCCCCACGAAGCAAACAACAC; 1286 bp deletion) (Wormbase), *flp-19* (CACATTTCACCGGTTTGTCG/TACTTGAACCGAAATTTACT; 1946 bp deletion), *flp-20* (TTACTTTTAATACGTCTAAT/CCATTCATTTTAAAAGAATT; 1343 bp deletion), *flp-21(pk1601)* (GAAAAAAGAAGAACCTACAT/TCAGAAAAAAGTAAACTAAT ; 464 bp deletion) [14], and *flp-21(ok889)* (GAGCAGTAGATTTTTCAAGT/TTCCTACCAAAGCCGAGCCGA; 1786 bp deletion) (Wormbase).

At least 31 genes encode 72 potential FLP peptides [6,8,9,38]; by cDNA screening all of these genes are expressed [6,8]Wormbase) and 56 of the predicted peptides have been biochemically isolated [39,40,41,42,43,44,45,46,47,48,49]. These genes are expressed in neurons involved in multiple behaviors, including locomotion, reproduction, aggregation, and pharyngeal pumping [22]. However, how these FLP peptides modulate these neural circuits are still unclear. To examine the function of the different FLP peptides, we initiated a project to isolate mutants for each of the *flp* genes. We screened several mutagenized libraries for *flp* deletion mutants [32]. Because many of the *flp* genes encode multiple peptides (Fig 1), we reasoned that a deletion of the *flp* coding region was more likely to result in a null mutation. Eleven *flp* mutants were the first to be isolated: *flp-3*, *4*, *6*, *8*, *9*, *10*, *12*, *18*, *19*, *20*, and *21*. The complete coding region for *flp-3*, *6*, *8*, *19*, *20*, and *21(ok889)* were deleted in the isolated mutants; the peptide coding region was deleted in the *flp-4*, *9*, and *12* mutants and the promoter region with peptide or non-peptide coding regions were deleted in the *flp-1*, *10*, *18*, and *21(pk1601)* mutants (Fig 1). We also included *daf-10 flp-1(yn2)* in our assays; the *yn2* deletion knocks out two genes, *flp-1* and the neighboring *daf-10*, and will be referred to as *flp-1* hereafter [11]. The 12 *flp* mutants were examined for locomotory and egg-laying behavior.

Because a single *flp* gene can be expressed in multiple types of neurons (e.g., sensory and motoneurons or sensory and interneurons), it is difficult to categorize the *flp* genes into distinct groups. Among the *flp* genes being analyzed, a few, *flp-4*, *6*, and *8*, are expressed predominantly in sensory neurons, but multiple types of sensory neurons. The more common example is that *flp* genes are expressed in many types of sensory neurons as well as other types of neurons. For instance, many of the examined *flp* genes expressed in chemosensory neurons (*flp-4*, *6*, *10*, *20*, and *21*) [50] are also expressed in other types of sensory neurons, such as gentle mechanosensory (*flp-4*, *8*, *12*, and *20*) [51], harsh mechanosensory (*flp-4*) [52], thermosensory (*flp-6*) [53], and/or oxygen/carbon dioxide sensory (*flp-3*, *4*, *6*, *8*, *10*, *19*, and *20*) [54,55,56] neurons [22] as well as interneurons (*flp-6*, *8*, *10*, and *20*) or motoneurons (*flp-21*). Only two of the examined *flp* genes are expressed predominantly in interneurons (*flp-1* and *18*) or in sensory, motor, and interneurons (*flp-12* and *19*). The expression pattern of one gene among the isolated mutants, *flp-9*, has not been determined.

### The role of FLPs in locomotion

Swimming initiates in *C*. *elegans* when animals are immersed in liquid. The body flexures result from the alternating waves of excitation/inhibition of the body wall muscles mediated by the excitatory cholinergic A and B motoneurons [23,26] and the inhibitory GABAergic D motoneurons [23,27]. Multiple sensory neurons synapse directly or indirectly onto the command interneurons PVC and AVB, which synapse onto the ventral cord motoneurons VB and DB for forward movement, and onto the command interneurons AVA, AVD, and AVE, which synapse onto the ventral cord motoneurons VA and DA for backward movement [23,51]. The A and B neurons synapse onto the ventral cord inhibitory motoneurons VD and DD to ensure proper body flexures [23]. Hence, locomotion is the integrated output of multiple sensory inputs. Because the *flp* genes are expressed in a variety of neurons, we expected that altering many *flp* genes would affect swimming rates. None of the *flp* genes examined are expressed in the ventral cord motoneurons. Two *flp* genes, *flp-1* and *18*, are expressed in the locomotory command interneurons.

**Fig 2:**
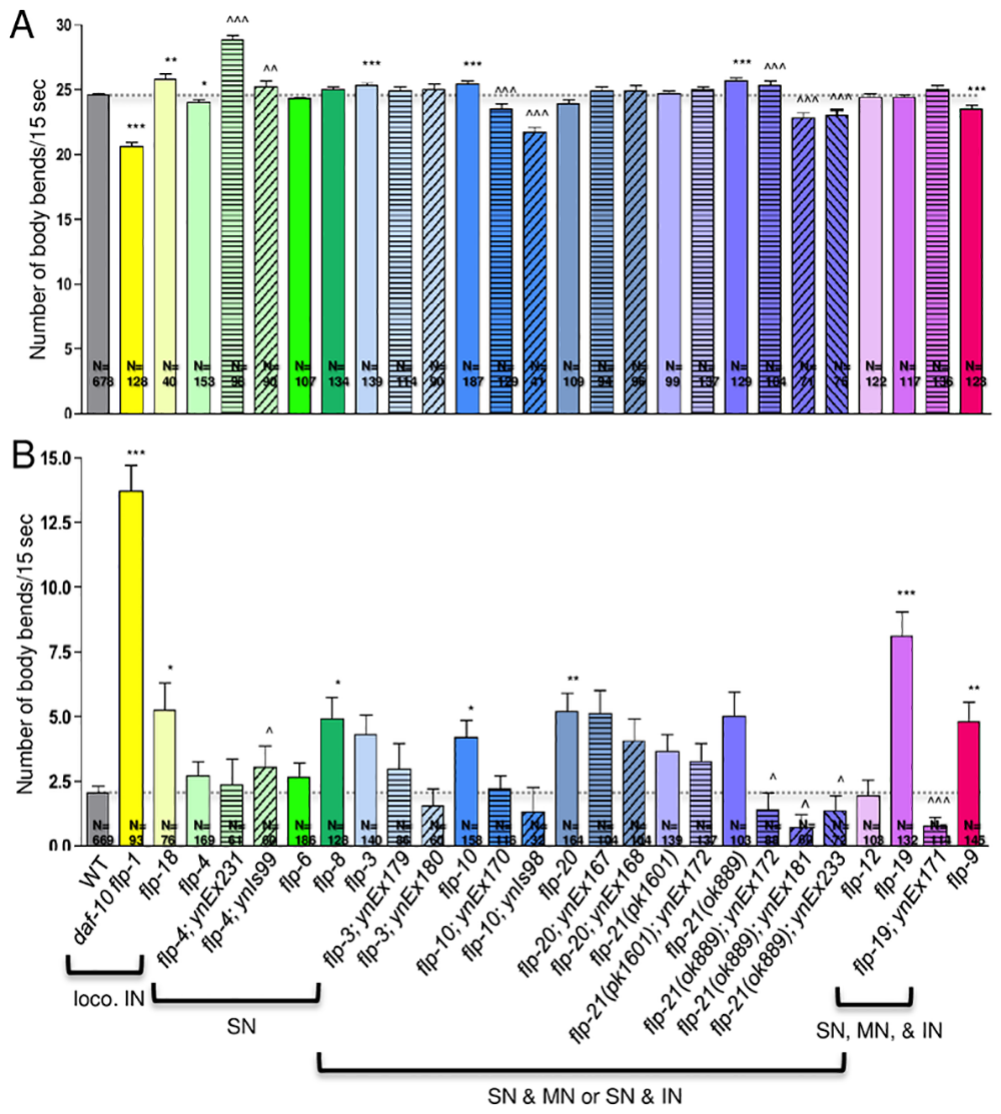
Loss of *flp* genes results in defects in locomotion. (A) Disruption of multiple *flp* genes affects swimming rate. One day hermaphrodite adults were scored for the number of body bends per 15 seconds in physiological M9 buffer. (B) Many *flp* mutants show resistance to serotonin-induced inhibition on swimming locomotion. One day hermaphrodite adults were scored for the number of body bends per 15 seconds in physiological M9 buffer with 12.9 mM serotonin. Mean and SEM shown. N = number of animals examined; each animal was tested three times and the values averaged for that animal. At least three trials were performed for each strain. Mutants are color-coded and were grouped according to predominant expression pattern; loco. IN, *flp* expression in locomotory interneurons; SN, *flp* expression predominantly in sensory neurons; MN, *flp* expression predominantly in motoneurons; IN, *flp* expression predominantly in interneurons; solid bars indicate mutants, patterned bars indicate mutants containing corresponding genomic fragment; transgenic animals also indicated by [], Ex indicates non-integrated array, Is indicates integrated array; *myo-2*p::*GFP* and/or *sur-5*::*GFP* were used as the transgenic marker (see Table 1). *, p<0.05, **, p<0.01, ***, p<0.001 significantly different from wild type, Mann-Whitney test; ^, p<0.05, ^^, p<0.01, ^^^, p<0.001 significantly different from mutant, one-way ANOVA, Neuman Keuls posthoc test.

Swimming behavior can be quantified by counting the number of body bends in 15 seconds (Fig 2A; Table 2). Wild-type animals have a swimming rate of 24.66±0.10 body bends/15 sec (n = 678). Loss of *flp-1* or *18*, whose expression patterns include the command interneurons, caused a decreased swimming rate in *flp-1* (20.64±0.30 body bends/15 sec, n = 128), but an increased swimming rate in *flp-18* (25.80±0.43 body bends/15 sec, n = 40) mutants, indicating that loss of these peptides causes a downstream effect in the locomotory circuit. Among the *flp* genes whose expression patterns are predominantly in sensory neurons, only *flp-4* mutants showed defects in swimming rate (24.00±0.24 body bends/15 sec, n = 153). Several mutants whose expression patterns include multiple types of neurons displayed hyperactive swimming, such as *flp-3* (25.27±0.21 body bends/15 sec, n = 139), *10* (25.43±0.27 body bends/15 sec, n = 187), and *21(ok889)* (25.74±0.17 body bends/15 sec, n = 129) mutants. *flp-9* mutants (23.55±0.24 body bends/15 sec, n = 123) showed a significantly decreased swimming rate. Hence, knockout of multiple *flp* genes expressed in a variety of neurons, but not in the locomotory motoneurons, affect swimming behavior.

**Table 2:** Multiple *flp* genes affect swimming and egg-laying behavior.

Genotype	Swimming Rate/15 sec.	Swimming Rate/15 sec. with 5-HT	Egg-laying Rate/hr	Egg Retention
Wild type (N2)	24.66±0.10 (n = 678)	2.07±0.23 (n = 669)	5.77±0.18 (n = 658)	13.28±0.29 (n = 701)
**Expressed mainly in sensory neurons**				
*flp-4(yn35)*	24.00±0.24 (n = 153)*	2.71±0.57 (n = 169)	5.49±0.46 (n = 116)	15.64±0.62 (n = 159)
*flp-4; ynEx231 [flp-4]#1*	28.81±0.43 (n = 98)^^^	2.33±1.02 (n = 61)	2.30±0.40 (n = 61)^^^	12.27±0.77 (n = 67)
*flp-4; ynEx178 [flp-4]#2*	28.37±0.39 (n = 93)^^^	5.14±1.34 (n = 60)	3.42±0.51 (n = 60)^^	10.17±1.00 (n = 64)^^^
*flp-4; ynIs99 [flp-4]#3*	25.18±0.52 (n = 90)^	3.07±0.81 (n = 60)^	4.83±0.47 (n = 60)	15.90±1.01 (n = 30)
*flp-6(pk1593)*	24.28±0.18 (n = 107)	2.65±0.54 (n = 186)	4.04±0.36 (n = 106)***	7.17±0.29 (n = 174)***
*flp-8(pk360)*	24.99±0.24 (n = 134)	4.92±0.83 (n = 128)*	7.43±0.60 (n = 83)*	13.19±0.60 (n = 170)
**Expressed mainly in interneurons**				
*daf-10 flp-1(yn2)*	20.64±0.30 (n = 128)***	13.68±1.01 (n = 93)***	2.82±0.44 (n = 67)***	19.59±0.92 (n = 108)***
*flp-18(tm2197)*	25.80±0.43 (n = 40)**	5.25±1.05 (n = 76)*	6.26±0.52 (n = 76)	12.37±1.02 (n = 41)
**Expressed in sensory and polymodal neurons**				
*flp-3(pk361)*	25.27±0.21 (n = 139)***	4.33±0.75 (n = 140)	5.80±0.46 (n = 120)	12.36±0.44 (n = 227)
*flp-3;ynEx179 [flp-3]#1*	24.96±0.23 (n = 114)	1.57±0.63 (n = 86)^	2.86±0.37 (n = 86)^^^	7.05±0.48 (n = 86)^^^
*flp-3; ynEx180 [flp-3]#2*	25.02±0.37 (n = 90)	2.97±1.00 (n = 60)	7.08±0.60 (n = 60)	9.67±0.73 (n = 60)^
**Expressed mainly in sensory and interneurons**				
*flp-10(pk367)*	25.43±0.27 (n = 187)***	4.21±0.66 (n = 158)*	2.16±0.20 (n = 215)***	8.29±0.31 (n = 214)***
*flp-10; ynEx169 [flp-10]#1*	23.29±0.44 (n = 88)^^^	1.28±0.48 (n = 102)	6.37±0.75 (n = 84)^^^	26.45±1.11 (n = 107)^^^
*flp-10; ynEx170 [flp-10]#2*	23.50±0.39 (n = 129)^^^	2.20±0.51 (n = 116)	4.41±0.54 (n = 82)	26.16±1.27 (n = 97)^^^
*flp-10; ynIs92 [flp-10]#3*	23.60±0.32 (n = 127)^^^	4.93±0.98 (n = 90)	9.05±0.92 (n = 60)^^^	29.79±1.17 (n = 106)^^^
*flp-10; ynIs93 [flp-10]#4*	19.72±1.09 (n = 65)^^^	9.46±1.52 (n = 63)^^^	1.54±0.42 (n = 33)	20.25±1.62 (n = 51)^^^
*flp-10; ynIs94 [flp-10]#5*	23.11±0.22 (n = 92)^^^	1.15±0.68 (n = 57)	5.96±0.77 (n = 57)^^^	36.53±1.42 (n = 30)^^^
*flp-10; ynIs95 [flp-10]#6*	24.28±0.46 (n = 42)	0.59±0.42 (n = 59)^	2.83±0.46 (n = 59)	33.10±2.82 (n = 30)^^^
*flp-10; ynIs98 [flp-10]#7*	21.75±0.37 (n = 41)^^^	1.32±.98 (n = 32)	6.19±0.89 (n = 32)^^	18.33±1.06 (n = 112)^^^
*flp-20(pk1596)*	23.89±0.34 (n = 109)	5.19±0.72 (n = 164)**	0.17±0.05 (n = 162)***	32.69±1.05 (n = 240)***
*flp-20;ynEx167 [flp-20]#1*	24.88±0.35 (n = 94)	5.13±0.87 (n = 104)	0.91±0.36 (n = 74)^^	27.82±1.59 (n = 61)^
*flp-20; ynEx168 [flp-20]#2*	24.93±0.36 (n = 96)	4.07±0.84 (n = 104)	0.69±0.16 (n = 74)^	26.16±0.99 (n = 143)^^^
**Expressed mainly in sensory and motorneurons**				
*flp-21(pk1601)*	24.70±0.26 (n = 99)	3.63±0.69 (n = 139)	3.40±0.37 (n = 112)***	18.78±0.76 (n = 156)***
*flp-21(pk1601); ynEx172 [flp-21]*	25.02±0.22 (n = 137)	3.26±0.68 (n = 137)	3.23±0.38 (n = 137)	11.02±0.72 (n = 85)^^^
*flp-21(ok889)*	25.74±0.17 (n = 129)***	5.00±0.97 (n = 103)	4.92±0.49 (n = 75)***	17.11±0.68 (n = 75)***
*flp-21(ok889);ynEx172 [flp-21]#1*	25.37±0.35 (n = 104)^^^	1.41±0.66 (n = 88)^	3.92±0.45 (n = 116)	10.00±0.55 (n = 90)^^^
*flp-21(ok889); ynEx181 [flp-21]#2*	22.86±0.37 (n = 71)^^^	0.71±0.50 (n = 60)^	4.63±0.65 (n = 60)	17.85±0.83 (n = 78)
*flp-21(ok889); ynEx233 [flp-21]#3*	23.04±0.36 (n = 75)^^^	1.38±0.60 (n = 72)^	4.15±0.44 (n = 72)	13.69±0.87 (n = 71)^^
**Expressed in sensory, interneurons, and motorneurons**				
*flp-12(n4902)*	24.44±0.27 (n = 122)	1.95±0.63 (n = 103)	4.41±0.46 (n = 91)**	19.75±0.84 (n = 183)***
*flp-19(pk1594)*	24.39±0.24 (n = 117)	8.10±0.95 (n = 132)***	3.89±0.46 (n = 93)***	16.46±0.79 (n = 138)**
*flp-19;ynEx171 [flp-19]#1*	25.06±0.27 (n = 136)	0.75±0.37 (n = 114)^^^	8.36±0.66 (n = 84)^^^	22.89±0.69 (n = 140)^^^
*flp-19; ynEx232 [flp-19]#2*	ND	1.87±1.16 (n = 30)^^^	2.37±0.58 (n = 30)	19.22±1.44 (n = 36)
**Expression pattern unknown**				
*flp-9(yn36)*	23.55±0.24 (n = 123)***	4.81±0.73 (n = 145)**	7.43±0.50 (n = 110)**	11.98±0.59 (n = 128)*

ND = not determined. [], indicates transgenic lines carrying the genomic region of the indicated gene; multiple independent lines were generated.

*, p<0.05,

**, p<0.01,

***, p<0.001 significantly different from wild type, Mann-Whitney test;

^^^, p<0.05,

^^^^, p<0.01,

^^^^^, p<0.001 significantly different from mutant, one-way ANOVA, Neuman Keuls posthoc test.

To determine whether these swimming defects were due to loss of the corresponding *flp* gene, we microinjected the genomic region corresponding to representative genes (*flp-3*, *4*, *10*, *19*, *20*, and *21*) into the respective mutants and assayed at least two independent transgenic mutant lines for swimming. *flp-4* mutants showed a slightly decreased swimming rate, but in a transgenic mutant *flp-4* line, a significant increase in the swimming rate was detected (28.81±0.43 body bends/15 sec, n = 98), suggesting that FLP-4 peptides potentiate swimming rate. *flp-10* and *flp-21(ok889)* mutants showed an increased swimming rate and transgene expression of *flp-10* and *flp-21* caused a significant decrease in the swimming rate, suggesting that FLP-10 and FLP-21 peptides decrease swimming rates.

The presence of serotonin reduces locomotion in wild-type animals [37] through activity of the NSM and HSN neurons [37,57]. The swimming rate, for example, drops to 2.07 ± 0.23 body bends/15 sec (n = 669) in wild-type animals in the presence of serotonin (Fig 2B, Table 2). Several *flp* mutants were insensitive to varying degrees to this serotonin-induced inhibition. For instance, *flp-8*, *9*, and *20* mutants showed slight, but significant resistance, whereas *flp-1* and *19* mutants showed significant resistance to the serotonin-induced inhibition (Fig 2B, Table 2). This resistance to serotonin-induced inhibition in *flp-19* mutants could be rescued by a *flp-19* genomic fragment, suggesting that FLP-19 peptides do not affect swimming rate, but inhibit the action of serotonin on swimming. *flp-20* mutants had only modest resistance to the serotonin-induced inhibition, and transgenic *flp-20* mutants exhibited slight, but not significant serotonin-induced inhibition, suggesting that FLP-20 peptides have a modest effect on the serotonergic circuit.

### Egg laying is highly sensitive to levels of different FLPs

The egg-laying motor circuit consists of the serotonergic/peptidergic (*flp-19*) HSN [22,58] and cholinergic/peptidergic VC motoneurons [26,29], both of which synapse onto the vulval muscles [23] whose contractions allow the release of eggs. Animals alternate between an active and inactive egg-laying state; this temporal switch is regulated by the HSN and VC neurons [59]. Egg-laying rate is sensitive to environmental conditions, such as food availability [28], hypertonic salt concentrations [37], and mechanical vibrations [60], and is coordinated with other behaviors, such as locomotion [28,57]. Hence, sensory input into the egg-laying circuit plays a significant role in determining egg-laying output. The mechanosensory neuron PLM and BDU interneuron [22] synapse directly onto the HSN neurons [23], and express the *flp-20* and *10* genes, respectively. Because egg laying is affected by multiple sensory stimuli, we expected that loss of multiple *flp* genes would also indirectly affect egg laying.

**Fig 3:**
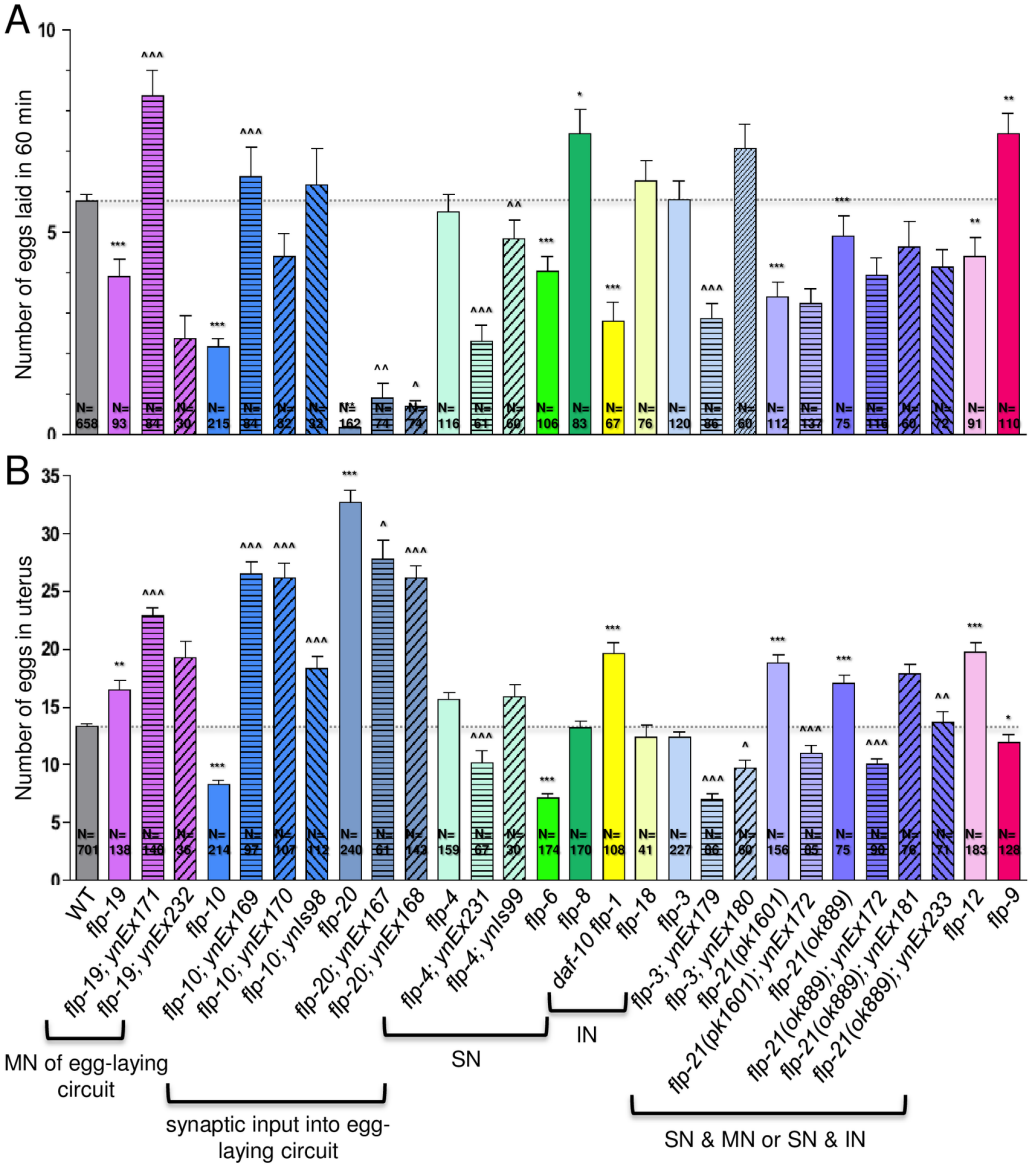
Several *flp* genes affect reproductive behavior. (A) One day hermaphrodite adults were allowed to lay eggs for 1 hour in the presence of serotonin. (B) Two day hermaphrodite adults were bleached to release eggs to determine the number of eggs in the uterus. Mean and SEM shown. N = number of animals examined; at least three trials were performed for each strain. Mutants are color-coded and were grouped according to predominant expression pattern; MN of egg-laying circuit, *flp* expression in motoneurons of egg-laying circuit; SN, *flp* expression predominantly in sensory neurons; IN, *flp* expression predominantly in interneurons; solid bars indicate mutants, patterned bars indicate mutants containing genomic fragment as indicated in brackets; *myo-2*p::*GFP* and/or *sur-5*::*GFP* were used as the transgenic markers (see Table 1). *, p<0.05, **, p<0.01, ***, p<0.001 significantly different from wild type, Mann-Whitney test; ^, p<0.05, ^^, p<0.01, ^^^, p<0.001 significantly different from mutant, one-way ANOVA, Neuman Keuls posthoc test.

Wild-type animals were stimulated by serotonin to lay 5.77±0.18 eggs (n = 658) per hour (Fig 3A; Table 2). We observed decreased egg-laying rates in most *flp* mutants. *flp-19* is expressed in chemo- and aerosensory neurons as well the HSN motoneurons within the egg-laying circuit. *flp-19* mutants showed a significant decrease in egg-laying rate (3.89±0.46 eggs; n = 93), which was rescued by transgene expression in one line (Fig 3A; Table 2). Whether this egg-laying decrease is due to loss of FLP-19 peptides from HSN or sensory input is unclear. For other *flp* genes that synapse directly onto the egg-laying circuit, both *flp-10* (2.16±0.20 eggs; n = 215) and *20* (0.17±0.05 eggs; n = 162) had significantly decreased egg-laying rates compared to wild type. The *flp-10* transgene rescued the egg-laying defect to varying degrees, suggesting that FLP-10 peptides potentiates egg-laying rate. Surprisingly, *flp-20* displayed an extremely low egg-laying rate, but presence of its transgene had only a slight, but significant, increase in the egg-laying rate (Fig 3A; Table 2). For *flp* genes expressed in sensory neurons, there was a variety of responses. *flp-6* (4.04±0.36 eggs; n = 106) and *21(pk1601)* (3.40±0.37 eggs; n = 112) mutants showed a significant decrease, which could not be rescued by the *flp-21* transgene, while *flp-8* mutants (7.43±0.60 eggs; n = 83) showed a significant increase in the egg-laying rate (Fig 3; Table 2). Loss of *flp-1*, which is primarily expressed in interneurons, also showed a significant decrease in egg-laying rate (2.82±0.44 eggs; n = 67). Loss of *flp-4* had no effect on egg laying, but its overexpression caused a decrease in egg laying, suggesting that release of FLP-4 peptides from sensory circuits causes an indirect inhibitory effect on egg laying.

### FLPs modulate the number of eggs retained in the uterus

The altered egg-laying rate in the *flp* mutants could be caused by a number of factors, including a decreased number of eggs generated, an incorrect sensing of the number of eggs in the uterus, or a failure of egg release due to muscle insensitivity to serotonin. We examined whether the altered egg-laying rates were due to a decreased pool of eggs available to be released or an increased retention of eggs in the uterus. We and others [61] have found that wild-type animals have 10–15 eggs in their uterus at any one time (two day adults: 13.28±0.29 eggs (n = 701); Fig 3B; Table 2).

Loss of *flp-19* caused a decreased egg-laying rate and an increased egg retention rate (16.46±0.79 eggs; n = 138), indicating that the defective egg-laying rate in *flp-19* mutants is not due to decreased egg production, but due to decreased egg release. However, transgene expression of *flp-19* in mutants only exacerbated the egg retention defect (Fig 3B; Table 2), suggesting that FLP-19 peptides released from sensory or interneurons also affect reproduction. Loss of *flp-20*, which is expressed in neurons with direct synaptic input onto the egg-laying motor circuit, is similarly correlated. Among all the *flp* mutants examined, *flp-20* showed the lowest egg-laying rate and the highest number of retained eggs (32.69±1.05 eggs; n = 240), which could be partially rescued by a *flp-20* transgene. By contrast, *flp-10*, which is also expressed in a neuron with synaptic input into the egg-laying motor circuit, had a decreased egg-laying rate, and showed a decreased number of eggs retained in the uterus, suggesting that lack of egg production is responsible for the decreased egg laying rate; transgene expression of *flp-10* in mutants caused an extremely high number of retained eggs in the uterus, indicating that FLP-10 peptides may influence activity of the sensor that determines the optimal number of eggs in the uterus or serotonergic input onto vulval muscles. Among the other *flp* mutants, the inverse correlation between egg-laying rate and number of eggs retained in the uterus was seen in *flp-1*, *4*, *12*, and *21* mutants, again suggesting that decreased egg production is not responsible for the decreased egg-laying rate; the increased egg retention was rescued with the transgene in *flp-4* and *21* mutants. Like *flp-10* mutants, *flp-6* mutants showed a decreased egg-laying rate and a decreased number of eggs in the uterus (7.17±0.29 eggs; n = 174) (Table 2), suggesting that egg production may factor into the decreased egg-laying rate. These results reinforce our findings that multiple FLP peptides modulate the output of the egg-laying circuit.

## Discussion

Environmental stimuli are integrated through multiple sensory modalities to determine behavior. Hence, we expected that disruption of any neural circuit would ultimately have an effect on most behavioral outputs, such as locomotion and reproduction. In this paper, we explored the effects of FLP neuropeptides released from different neural circuits [23,62]. Because neuropeptides are generally co-localized with a small molecule transmitter [10,22] and have modulatory effects, we predicted that modulating levels of many of the FLP peptides would affect locomotion and reproduction and found this prediction to be correct.

Although FLP peptides are expressed in the locomotory motoneurons, none of the *flp* genes examined are expressed in the locomotory motoneurons. Five of the 11 *flp* mutants showed defective swimming rates, which could be at least partially rescued by re-introduction of the respective *flp* gene (Fig 2). The expression patterns of the affected *flp* genes were not specific for certain types of neurons. Instead, the affected mutants included genes expressed in interneurons (*flp-1* and *18*) (36, 39) and sensory neurons (*flp-4*, *10*, and *21*). *flp-8*, *10*, *12*, and *19* are expressed in the oxygen sensor neurons BAG and/or URX; however, only loss of *flp-10* affected swimming rates. Similarly, *flp-4*, *6*, *10*, and *20* are expressed in the chemosensory ASE neuron, but only *flp-4* and *10* knockouts showed modulated swimming rates (Table 2). *flp-4* is also expressed in one mechanosensory neuron, but other *flp* genes expressed in mechanosensory neurons (*flp-8*, *12*, and *20*) showed no change in swimming rate. Because *flp-4*, *10*, and *21* are expressed in so many different types of sensory neurons, it is difficult to ascribe their effects on swimming to a particular circuit. However, regulation of neuropeptide levels must be under very tight homeostatic control, because loss of many *flp* genes affect swimming rates and, conversely, transgene overexpression of *flp* genes also affect swimming rate. For instance, *flp-4* overexpression increased swimming rate, while *flp-10* and *21* overexpression decreased swimming rate, supporting the indirect role of these peptides in modulating swimming behavior. Among the characterized FLP peptides, many bind multiple receptors and the receptors through which these peptides signal show widespread expression. For instance, FLP-18 and 21 peptides bind at nM affinities to NPR-1, 3, 4, 5, 6, 10, and 11 [13,14,16]; for the NPR receptors that have been characterized, NPR-1 and 4 show widespread expression [16,63], making it difficult to draw conclusions as to which downstream neurons and circuits are being activated by the FLPs to affect locomotion.

The transgenic lines often gave a wide variety of responses, which we attribute to variation due to the mosaic nature of transgene expression. Unlike other organisms where the transgene is inserted into the genome, *C*. *elegans* transgenes are present as extrachromosomal arrays, which can be lost at any cell division. Hence, even within one non-integrated *C*. *elegans* transgenic line responses are variable, so multiple lines were analyzed. In addition, varying lengths of the promoter and 3’UTR regions (Table 1) for the different genomic fragments were used; longer lengths of these regions may contribute to better stability of the transcript, which could have resulted in better and/or less variable responses.

Within the reproductive motor circuit, none of the examined *flp* genes are expressed in the VC motoneurons or AVF command interneuron [22], but *flp-19* is expressed in the HSN motoneurons, the critical neuron regulating the active egg-laying state [59]. Not surprisingly, modulating levels of FLP-19 peptides affected egg-laying rates. Loss of *flp-19* decreased egg-laying rates, which could be rescued by re-introduction of FLP-19 peptides, suggesting that FLP-19 peptides released from HSN potentiates egg laying. No FLP-19 receptor has been identified thus far. Similarly, *flp-10* is expressed in the BDU interneuron that synapses onto HSN, and again, not surprisingly, decreasing and increasing levels of FLP-10 peptide decreased or increased the egg-laying rate, respectively. The single FLP-10 peptide signals through the EGL-6 receptor, which is expressed by HSN neurons [15], suggesting that FLP-10 promotes egg laying by activating HSN. These results are in contrast to those of Ringstad and Horvitz [15], who reported that overexpression of *flp-10* inhibited egg laying, while knockout of *egl-6* had no effect on egg laying, as scored by the stage of the egg laid as opposed to egg-laying rate; in addition, these authors did not report *flp-10* expression in BDU neurons with their expression vector, which may account for our differing results. Ringstad and Horvitz [15] also reported that *flp-17* activity within the oxygen sensor BAG neuron inhibited egg laying. In addition to *flp-10* and *flp-17*, *flp-12* and *19* are expressed in the BAG neuron. Like *flp-10* and *19*, loss of *flp-12* also decreased the egg-laying rate. Because *flp-10* and *19* are expressed in multiple neurons within the egg-laying neural circuits, it is unclear from which neurons the peptides are exerting their effects on egg laying. Among other *flp* genes expressed in sensory neurons, loss of *flp-6*, *20*, and *21* decreased the egg-laying rate, while *flp-8* knockouts, as well as knockout of *flp-9*, whose expression pattern is unknown, showed an increased egg-laying rate; transgenic copies of *flp-20* and *21* in the mutants caused the converse phenotype, indicating that, as previously reported [61], multiple sensory stimuli affect egg-laying rate. RNAi knockdown of FLP-21 receptors NPR-3, 6, and 11 also decreased brood size [64], suggesting that FLP-21 signals through these receptors to modulate egg laying. The receptors through which FLP-6, 9, 12, 19, and 20 peptides signal have not been identified.

We hypothesized that if there was a problem with egg release, egg-laying rates would be inversely correlated to egg retention in the uterus. Specifically, we expected that animals that displayed high egg-laying rates would have low numbers of eggs in the uterus and, conversely, animals with low egg-laying rates would have high numbers of eggs retained in the uterus. This inverse correlation was seen among several mutants. *flp-1*, *12*, *19*, *20*, and *21* mutants showed low egg-laying rates and high numbers of eggs in the uterus, suggesting that eggs were being generated, but were not being released, perhaps because the neurons were not signaling to the muscles properly or the muscles were not responsive to serotonin. Conversely, *flp-9* mutants showed an increased egg-laying rate and a decreased number of eggs retained, suggesting that eggs were laid as soon as they were generated and *flp-9* is involved in the timing of egg release. There were also mutants, such as *flp-4*, whose egg-laying rate was unaffected, yet it retained a significant number of animals in its uterus; this egg retention defect was rescued with a wild-type copy of *flp-4* (Table 2) and may indicate a disruption of a sensor that regulates the optimal number of eggs in the uterus. Several mutants showed a positive correlation in egg-laying rate and egg retention. For instance, *flp-3*, *6*, *10*, and *19* mutants showed significant decreases in the egg-laying rate as well as a decreased number of eggs retained, suggesting that there is a disruption in egg formation. Hence, the FLP peptides affect reproductive behavior in a multitude of ways.

## Conclusions

We examined the effects of loss of FLP neuropeptides on locomotory and reproductive behavior. Because environmental stimuli activate multiple sensory systems that are integrated to determine locomotory and reproductive behavior, we have found that any perturbation of sensory or motor systems, such as by altering the levels of neuropeptide signaling, will modulate these behaviors. Furthermore, we have not explored, because of technical difficulties, whether neuropeptides exert their effects extrasynaptically, such that neuropeptides released within the nerve ring may affect neural circuits for which they do not have a direct synaptic connection. Our work has shown the interconnections between all neural circuits in the control of critical behaviors in an organism. Nematodes have the largest known family of FLPs in the animal kingdom [65]. The role of each specific FLP peptide in locomotion and reproduction awaits further studies.
